# Computational modeling of sphingolipid metabolism

**DOI:** 10.1186/s12918-015-0176-9

**Published:** 2015-08-15

**Authors:** Weronika Wronowska, Agata Charzyńska, Karol Nienałtowski, Anna Gambin

**Affiliations:** Faculty of Biology University of Warsaw, Warsaw, Poland; Bioinformatics Laboratory, Mossakowski Medical Research Centre, Polish Academy of Sciences, Warsaw, Poland; Institute of Computer Science Polish Academy of Sciences, Warsaw, Poland; Institute of Fundamental Technological Research, Polish Academy of Sciences, Warsaw, Poland; Institute of Informatics, University of Warsaw, Warsaw, Poland

**Keywords:** Sphingolipid metabolism, Kinetic model, Sensitivity analysis

## Abstract

**Background:**

As suggested by the origin of the word, sphingolipids are mysterious molecules with various roles in antagonistic cellular processes such as autophagy, apoptosis, proliferation and differentiation. Moreover, sphingolipids have recently been recognized as important messengers in cellular signaling pathways. Notably, sphingolipid metabolism disorders have been observed in various pathological conditions such as cancer and neurodegeneration.

**Results:**

The existing formal models of sphingolipid metabolism focus mainly on *de novo* ceramide synthesis or are limited to biochemical transformations of particular subspecies. Here, we propose the first comprehensive computational model of sphingolipid metabolism in human tissue. Contrary to the previous approaches, we use a model that reflects cell compartmentalization thereby highlighting the differences among individual organelles.

**Conclusions:**

The model that we present here was validated using recently proposed methods of model analysis, allowing to detect the most sensitive and experimentally non-identifiable parameters and determine the main sources of model variance. Moreover, we demonstrate the usefulness of our model in the study of molecular processes underlying Alzheimer’s disease, which are associated with sphingolipid metabolism.

**Electronic supplementary material:**

The online version of this article (doi:10.1186/s12918-015-0176-9) contains supplementary material, which is available to authorized users.

## Background

Sphingolipids (SL) are a class of complex lipids with a sphingoid base (Sph) [1]. Modifications of this basic structure that consist in the addition of an amide-linked fatty acid or phosphorylation lead to the formation of bioactive sphingolipids such as ceramide (CER), ceramide-1-phosphate (C1P), sphingosine-1-phosphate (S1P) or sphingomyelin (SM) [2, 3]. Ceramide is a recognized branching point in the metabolism of various sphingolipids subspecies. There are three major pathways of ceramide synthesis. In *de novo* synthesis pathway ceramide is created from less complex molecules [4]. The second pathway is the catabolism of complex sphingolipids, mainly sphingomyelin [5]. Ceramides can also form through the breakdown of complex sphingolipids that are ultimately broken down into sphingosine in the acidic environment of the lysosome. In this pathway, known as *salvage pathway*, sphingosine is then reused, it is reacetylated to form ceramide again [6]. At the same time, ceramide may serve as a substrate in the synthesis of SM, C1P, and Sph which, in turn, can be phosphorylated to S1P [7–11]. For a long time, sphingolipids were believed to serve mainly structural purposes and have only been recognized as important messengers in cellular signaling pathways [12, 13] in the last two decades.

A notable body of work has been devoted to studying the influence of sphingolipid metabolism on cellular fate: autophagy, apoptosis, proliferation or differentiation [14, 15]. Importantly, individual sphingolipid species appear to have an antagonistic effect on cell growth and survival. The dynamic balance between proapoptotic (e.g. CER and Sph) and antiapoptotic (prosurvival) molecules (e.g. S1P and C1P) is termed *sphingolipid rheostat* [16]. Disruptions in the metabolic pathways involved in the regulation of this balance are believed to underlay various diseases. Indeed, sphingolipids are known to have critical implications for the pathogenesis and treatment of diverse conditions such as cancer [17–20] and neurodegenerative disorders (e.g. Alzheimer’s disease) [21–25].

### Related research

Formal modeling appears to be an excellent tool to predict the response of a system to a wide range of both external and internal factors in different scenarios. However, due to the complexity of the sphingolipid metabolome and the paucity of data, not much research has been performed in the field of computational sphingolipidome modeling. Only few models of SL metabolism are available in literature. The model provided by Vasquez et al. [26] refers to *de novo* ceramide synthesis in yeast. It contains all essential elements of ceramide synthesis from nonsphingolipid metabolism. However, no further steps involving the recycling of ceramides and other more complex sphingolipids (such as the SM catabolic pathway and *salvage pathway*) are considered. The model proposed by Gupta et al. [27] describes the C16-branch of sphingolipid metabolism in RAW264.7 cells. An advantage of this model is that it combines the lipidomics and transcriptomics data provided by the LIPID MAPS Consortium. However, the model applied here is restricted to the closest metabolites of C16 ceramide. None of the proposed models contain cell compartmental division despite the fact that ceramide metabolism is known to differ depending on the cell compartment such as the mitochondrion, the nucleus and the cell membrane. Therefore, we found it appealing to create a computational model for the metabolism of complex sphingolipids in human tissues.

### Our results

We propose a formal model of regulatory processes that contain sphingolipid metabolism pathways. Computational modeling is based on ordinary differential equations (ODEs) that describe the evolution of species concentration. The kinetics of our model is based on Mass Action Law (MAL) for molecular transport reactions and the Michaelis Menten (MM) approach for enzymatically catalyzed reactions. The model also covers the potential inhibitory effects of some species on the synthesis of other species.

To the best of our knowledge, this is the first computational model of sphingolipid metabolism that includes compartmentalization based on the typical structure of a nondifferentiated eukaryotic cell. Reaction parameters were estimated on the basis of publicly available literature data and some default assumptions based on experience with Biochemical Systems Theory [28], whereas the initial concentrations of particular sphingolipd species in each organelle were obtained from the LIPID MAPS database [29]. To validate our model, we applied both standard and novel methods of analysis, i.e. local sensitivity analysis [30], variance decomposition [31] and clustering of model parameters based on sensitivity indices [32]. Finally, we demonstrate the utility of our model to study the molecular events underlying Alzheimer’s disease (AD). The proposed model provides comprehensive, functional integration of experimental data and will contribute to the understanding of the interrelationships between sphingolipid metabolism and various diseases that remain elusive. Moreover, this is the first time that two recently published methods of computational model analysis (i.e. variance decomposition [31] and sensitivity clustering [32]) are applied in a medium-size realistic biochemical model.

## Results and discussion

### Model of sphingolipid metabolism

**Fig. 1:**
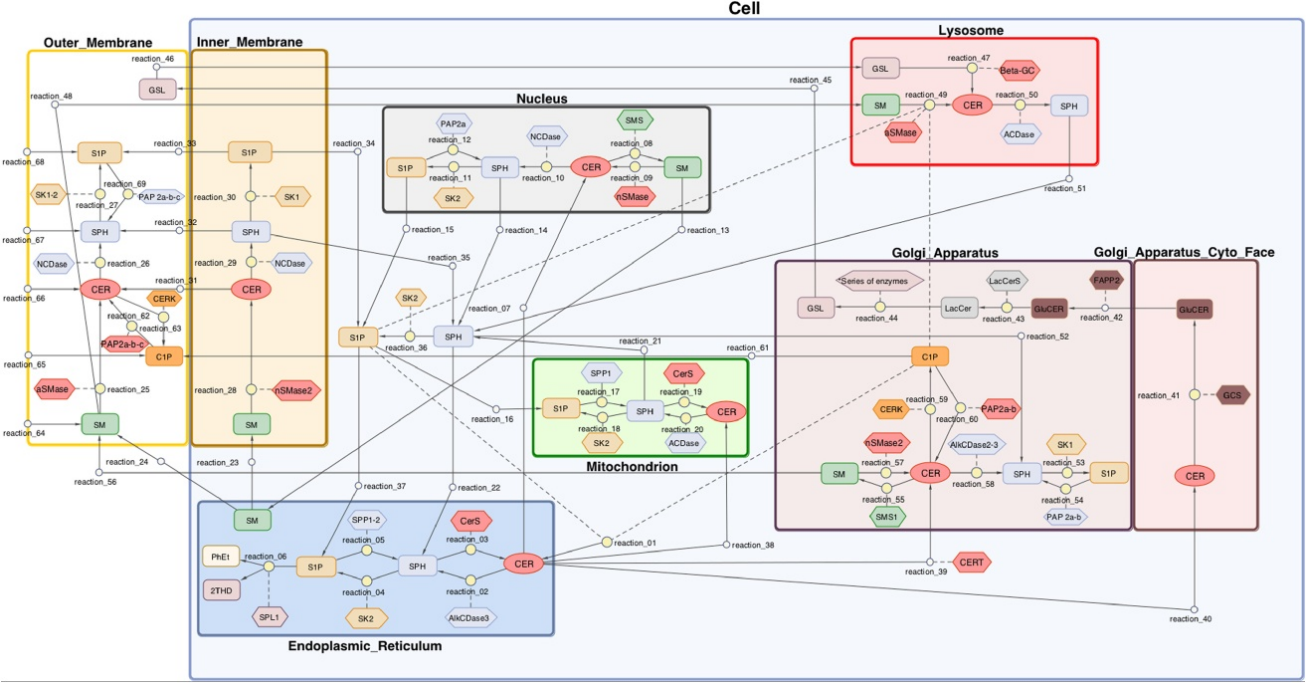
The sphingolipid metabolism diagram. Network of the SL metabolism system. Diagram was generated in Matlab Simbiology software. The full model contains 69 reactions, 39 modeled species and 37 reaction catalyzing enzymes. Oval boxes denote reacting molecule species, diamond boxes denote enzymes, circles denote reactions (small circles – transport, bigger circle metabolic reaction). Solid lines connect reactants with reaction, short dash lines connecting diamonds to reactions denote enzymatic catalyzing influence. Long dash lines connecting ovals to reactions denote inhibition

Our model captures all essential elements of the complex network of sphingolipid metabolism excluding *de novo* ceramide synthesis which has been described by Vasquez et al. [26]. It illustrates the general behavior of selected subspecies in unspecified human tissue in nine subcellular compartments. These compartments represent the following organelles or their parts: the outer and inner layer of the cell membrane, the cytoplasm, the endoplasmic reticulum, the cytoplasmatic and lumenal face of the Golgi apparatus, the nucleus, the mitochondrion and the lysosome. Our model includes 69 reactions of molecular transport and biochemical transformation (Fig. 1).

#### Transport

We applied the Mass Action Law principle to describe transport kinetics. Particular equations simulate different transport pathways which are determined by the specific biophysical properties of particular sphingolipids [33]. It is worth highlighting that most of these molecules [i.e. CER, SM and glycosphingolipids (GSL)] are restricted to biological membranes. These can be transported between organelles only in the form of complexes with lipid transfer proteins [34], e.g. the CER transfer (CERT) protein binds to CER. In addition, sphingolipids may change their location in the form of vesicles, i.e. as an integral part of biological membranes [35]. For example, the translocation of SM and GSL from the Golgi apparatus to the outer membrane is associated with exocytosis. Furthermore, SL may move into the lysosome when the endocytosis complex is formed. SL species may also diffuse along the membranes of interlinked organelles, as is the case with ceramide that floats between the endoplasmic reticulum and the nucleus [36]. On the other hand, Sph, S1P and C1P are sufficiently hydrophilic to diffuse freely from membranes to the cytosol, and similarly from the outer membrane to the external environment [37]. However, it has been reported that the transport of C1P from the Golgi apparatus to other cellular compartments may also occur in association with specific transporter proteins such as C1P transfer protein (CPTP) [38]. Water solubility also determines a molecule’s ability to flip between membrane leaflets. CER has a relatively rapid flip rate and Sph is sufficiently amphipathic to move between membrane layers [39, 40]. Finally, S1P requires specific lipid transporters to traverse membranes [41, 42]. Complex sphingolipids are unable to cross membranes without the aid of specific flippases such as four-phosphate adaptor protein 2 (FAPP2) which draws glucosylceramide from the outer surface to the inner surface of Golgi cisterns [43].

#### Ceramide synthesis and degradation

The majority of reactions depicted in Fig. 1 are enzymatic. The Michaelis Menten model and simplified kinetics were applied to describe different pathways of synthesis and degradation of the selected SL species, including ceramids. There are three major pathways of ceramide synthesis. CER synthesis via *de novo* pathway is described as the inflow of these molecules into the endoplasmic reticulum. CER may also by generated through the acetylation of Sph. This reaction is catalyzed by different types of ceramide synthases (CerS) [44] and is the final step of the *salvage pathway* [6]. Notably, the endoplasmic Sph metabolized in this pathway may be generated from the degradation of S1P which is catalyzed by specific phosphatases (SPP1 and SPP2) [45] or from the lysosomal degradation of complex SL species. This pathway is initiated by acidic sphingomyelinase (aSMase) and is critical to maintain proper concentrations of cellular SL [46, 47]. In addition to the abovementioned endoplasmic route of CER synthesis, a similar subset of reactions occcurs in the mitochondria. The reactions of mitochondrial SL metabolism have not yet been completely understood. In particular, enzyme specificity and the values of reaction rate parameters are often unknown [48, 49].

The third route of CER synthesis is through the hydrolysis of SM. The enzymes responsible for catalyzing these reactions, sphingomyelinases (SMases) are classified into three categories based on their optimum pH values and subcellular distribution. The degradation of SM is essential for the homeostasis of cell membranes; it has also been reported to be strongly associated with stress induced apoptosis [14, 50, 51].

Finally, we describe CER hydrolysis. Ceramidases CDase, seven of which have been described in humans, catalyze the cleavage of fatty acids from CER which leads to the production of Sph [52].

#### Synthesis of complex SL

The most complex SL are SM and GSL which are even more diverse. Although some enzymes responsible for the synthesis of these complex SL have been detected in e.g. the nucleus, this pathway is mainly localized in the Golgi apparatus. In both cases, the CER is used as a backbone molecule. However, its conversion into either SM or GSL depends on the transport pathway from the endoplasmic reticulum. A CER transported in the complex with a CERT protein moves into the cis-Golgi where SM is generated in a reaction catalyzed by an SM synthase [53, 54]. On the other hand, CER must move into the trans-Golgi via a vesicle-dependent pathway to form GSL in a series of reactions [55].

#### S1P and C1P metabolism

Our model includes reactions of CER and Sph phosphorylation. The resulting S1P and C1P, unlike CER and Sph, promote cell growth and have anti-apoptotic properties [16, 56]. The effect of these metabolites is regulated by the activity of several enzymes: (i) ceramide kinase (CERK) responsible for the synthesis of C1P in the Golgi apparatus and the plasma membrane [57]; (ii) sphingosine kinases (SK1 and SK2) which catalyze the phosphorylation of Sph in different subcellular locations [58, 59]; and (iii) phosphatases that hydrolyze S1P and C1P. Phosphatases include both lipid phosphate phosphatases of broad specificity [Phosphatidic acid phosphatase types 2a (PAP2a), 2b (PAP2b) and 2c (PAP2c)] and S1P specific phosphatases (SPP1 and SPP2) [45, 60]. All of these enzymes with their isoforms differ in substrate specificity, optimum pH values and subcellular localization. Our model illustrates the majority of their known properties. For detailed characteristics see the reviewed articles [7–11]. It is worth highlighting that both S1P and C1P have been identified as inhibitors of enzymes responsible for CER synthesis, such as acidic sphingomyelinase (aSMase) and serine palmitoyltransferase (SPT), the key regulatory enzyme of *de novo* synthesis pathway. In our model, inhibitory kinetics were used to describe this inhibitory activity of S1P (see Additional file 1: Table S1). Finally, our model includes the reaction of irreversible S1P degradation. Catalyzed by sphingosine-1-phosphate lyase (SPL1), this reaction of S1P hydrolysis to hexadecenal and phosphoetanolamine allows to remove the sphingoid base from the pool of SL metabolites [60, 61].

#### Model parameters

In conclusion, our model consists of 39 variables that represent molecular species concentrations (some of these are the same compounds but localized in different compartments, cf. Fig. 1). The metabolic reaction network covers 69 biochemical reactions (enzymatic and transport-related) between the reacting species. The model is implemented in the form of a system of 69 ordinary differential equations (ODEs) that model the dynamics of the reaction network. The 129 parameters of inhibition and reaction rates in the stationary state that represent the conditions of homeostasis constitute the relevant input of the model and are presented in Additional file 1: Table S1 while the 38 initial values of species concentrations are presented in Additional file 1: Table S2. To achieve conditions that resemble the intracellular environment during homoeostasis we stabilized species concentrations to the stationary state of the system. The initial values of lipid levels were obtained from the LIPID MAPS [26]. In the following part of this article, the model was validated by local sensitivity analysis, variance decomposition and clustering analysis.

### Computational validation of the model

Biochemical models are characterized by a substantially larger number of parameters relative to size of available experimental data. Therefore, the exact estimation of model parameters is highly difficult [62]. Thus, we used mathematical modeling to analyze the interrelationships between parameters and model dynamics. To verify the assumptions of our model, several methods were applied to obtain a broad view of the behavior of the modeled system in normal and stress conditions. Validation methods were based on recently proposed and classical approaches that engage exact mathematical methods.

#### Local sensitivity analysis

**Fig. 2:**
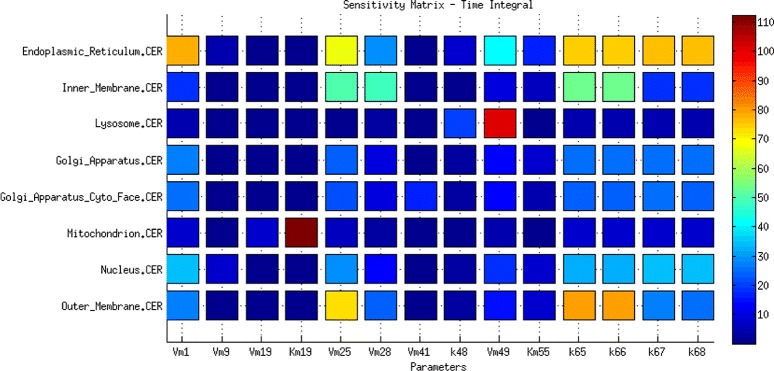
The local sensitivity analysis of the CER species to the highly significant parameters
- For the sphingosine species, the greatest instability behavior shows the mitochondrial Sph, that is highly sensitive to the model’s inflows parameters as well as the parameter of reaction catalyzed by the enzyme CerS in the mitochondrion. Moreover, mitochondrial Sph is sensitive to the SMase catalyzed reactions in membrane (Additional file 1: Figure S1). In contrast, concentration of Sph localized in cytosol is practically invariant to parameters.
- Mitochondrial S1P shows the greatest instability within the S1P species, not only for the model’s inflows parameters and CerS in mitochondrion, but also for parameters of reactions catalyzed by SMase in membrane and lysosome. For the other S1P species, the the mostly important is sphingosinokinase (SK) in the reticulum, inner membrane and nucleus, respectively. The cytoplasmic S1P is the most stable S1P species (Additional file 1: Figure S2).
- Sphingomyelin in the outer membrane is the dominant species within all other species; consequently, the exogenous inflow of SM by outer membrane is the most significant parameter for the SM species. This parameter does not play a noticeable role for other species that are sensitive for model’s inflows parameters by outer membrane (exogenous: C1P, CER, Sph and S1P). Another interesting feature is that the nuclear SM is the most stable within SM species (Additional file 1: Figure S3).

For the outcome of the local sensitivity analysis [30] performed for the system in stationary state homeostasis, see Fig. 2 and Additional file 1: Figures S1-S3. The following conclusions were drawn.

- The highest sensitivity indices among ceramide species were assigned to mitochondrial and lysosomal CER associated with the activity of ceramide synthase (CerS) in the mitochondrion and sphingomielynase (SMase) in the lysosome. The widest range of sensitivity was observed for CER in the endoplasmic reticulum, especially for the parameters of exogenous CER inflow through the outer membrane and endogenous CER via *de novo* synthesis in the endoplasmic reticulum. Notably, CER in the endoplasmic reticulum is also highly sensitive to the parameters of reactions catalyzed by SMase in the outer membrane. High sensitivity to the parameters of exogenous inflow of CER and C1P show membrane CER species, which are also sensitive to the membrane reactions catalyzed by SMases (Fig. 2).

#### Variance decomposition - homeostasis

**Fig. 3:**
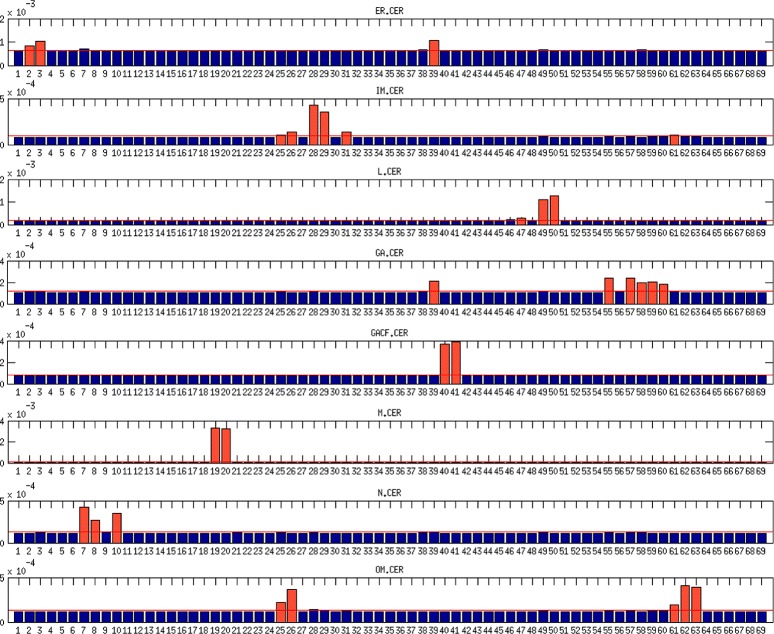
The variance decomposition of ceramide concentrations into components steaming from all model reactions. The red lines denote the average variance components of the investigated species. The red bars denote the variance components that exceeded the threshold of 110 % of average. The x-axis denotes reactions numbers and y-axis denotes the size of variance components
- Within the sphingosine species, the highest variance similarly to CER species falls to the mitochondrial Sph, whereas contrary to the mitochondrial CER for the mitochondrial Sph, all reactions’ noise components are near to average. Interesting is that two incident reactions connecting mitochondrial Sph with mitochondrial CER exceeded threshold and two other incident reactions connecting mitochondrial Sph with mitochondrial S1P are significantly below average. Similarly, nuclear and endoplasmic Sph have high and almost equally distributed noise with most significant incident reactions. For nuclear Sph the threshold was exceeded also by reactions non-incident with nuclear CER. For the membrane Sph species, highly influential reaction was the Sph membrane diffusion. For the inner membrane Sph except the incident reactions, the high noise components steams from reactions connected with outer membrane CER (between outer membrane SM and Sph). The outer membrane Sph significant reactions include transport reaction of C1P from Golgi apparatus to outer membrane and reactions connected with outer membrane CER and outer membrane S1P. For the lysosomal Sph, except incident reactions, the high noise component steams from reaction catalyzed by aSMase in lysosome. For the cytoplasmic Sph except incident reactions the threshold was exceeded by the reaction catalysed by Alkaline Ceramidase (AlkCDase) in Golgi apparatus. For Golgi apparatus Sph the noise was mainly decomposed by incident reactions.
- The highest variability within S1P species has the mitochondrial S1P; all its variance components showed near to average noise and none of the reactions exceeded the threshold of 110 %. Variance of all other S1P species steams principally from the incident reactions with an exception of cytoplasmic S1P, in which noise is generated mainly by lysosomal reaction catalyzed by aSMases.
- The SM species have the highest noise among all species and, contrary to most other species, the variance of SM species steams almost equally from all reactions.

The variance decomposition method enables to decompose noise associated with uncertainty of the modeled output into components related to different reactions [31, 63]. The application of this method to our model principally indicates the reactions corresponding to edges in Fig. 1 incident to investigated species as the highest noise generators. Nevertheless, some reactions were more significant for investigated species than other incident reactions, whereas for some other species, variances are distributed equally among all reactions. To find the distinctive reactions, we calculated the mean variance for each investigated species and set the threshold to 110 % of the mean variance. The results for CER species are depicted in Fig. 3 and those for Sph, S1P and SM are depicted in Additional file 1: Figures S4, S5 and S6, respectively.

- Within the ceramide species, the highest variance shows the mitochondrial and lysosomal CER. For CER, the threshold set on 110 % of average variance was exceeded only by the reactions catalyzed by ceramide synthase (CerS) and acid ceramidase (ACDase) in mitochondrion. The membrane CER species interact together; hence, for inner membrane CER not only incident reactions exceeded the threshold but also reaction incident with outer membrane CER catalyzed by aSMase. For the outer membrane CER, the highest variance component stems from the reactions incident with outer membrane C1P and transport reaction of C1P from Golgi apparatus to outer membrane. For the other CER species the highest variance is caused by the incident reactions.

#### Sensitivity-based parameter clustering (homeostasis)

Due to its complex structure, our model is perfectly suited to test the applicability of the new method of clustering mutually compensative parameters to detect mutual relationships between them [32]. In general, parameters sets are not pairwise independent and biochemical models are often sensitive to linear combinations of parameters, which makes them non-identifiable [62, 64].

**Fig. 4:**
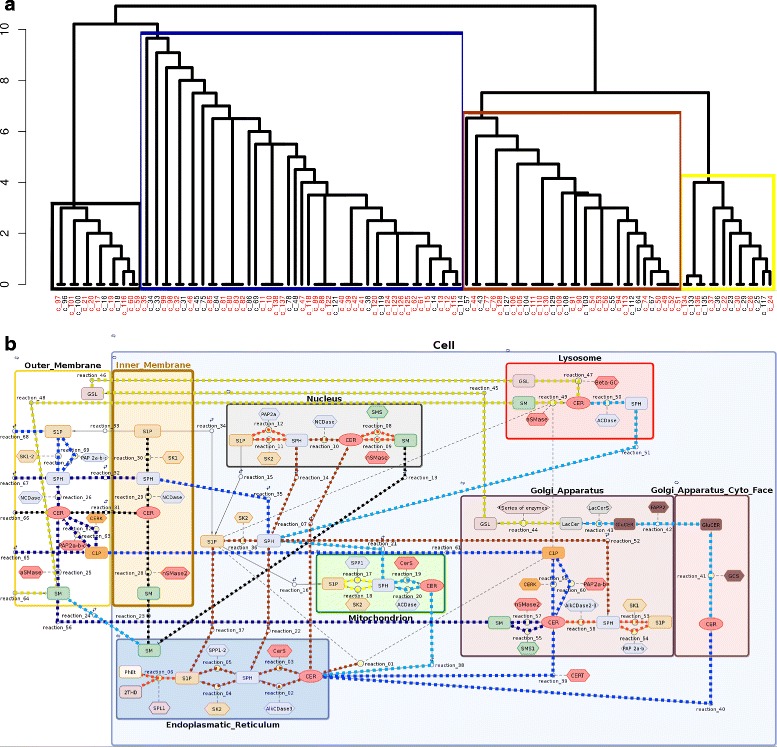
**a** Dendrogram obtained by hierarchical clustering of parameters based on their functional redundancy. Identifiability analysis yielded 37 unidentifiable parameters (marked in red).The labels and corresponding names of parameters are provided in Additional file 1. **b** Clusters of reactions induced by hierarchical grouping. The colors of connections between species are compatible with the colors of clusters in the dendrogram. Color intensity within the cluster corresponds with the level of redundancy between reaction parameters and other parameters in the cluster

Through sensitivity clustering of parameters (see Section Methods) we obtained a dendrogram where four clusters may be clearly distinguished (Fig. 4). These clusters may be interpreted as specific functional modules. Our results are compatible with the theoretical compartments recognized by Rao et al. [65], who presented the sphingolipid metabolism pathway as a combination of the following units: (i) the C1 compartment that represents the *de novo* biosynthesis of CER, (ii) the C2 compartment that reflects the conversion of CER into complex sphingolipids such as SM and GSL, (iii) the C3 compartment that represents the hydrolysis of SM to CER and (iv) the C4 compartment that reflects the conversion of CER into bioactive molecules such as C1P and S1P.

#### Ceramide phosphorylation

In our model, the brown cluster includes reaction parameters from the endoplasmic reticulum, the Golgi apparatus, the nucleus and the cytoplasm. The strong redundancy among parameters reflects their functional correlation. This cluster is primarily related to the conversion of Sph to S1P and vice versa. Beyond sphingolipid phosphorylation and dephosphorylation, it also contains reactions of sphingosine acetylation and CER deacetylation. This cluster corresponds to the C4 compartment described by Rao et al. [65] to some extent. However, our cluster fails to include reactions that occur in the cell membrane; these form part of the blue and green clusters. However, we found the reaction that reflects the endogenous inflow of ceramides into the endoplasmic reticulum (simulating the *de novo* synthesis pathway) to be a part of the brown cluster. Notably, our analysis shows that reactions in the endoplasmic reticulum are in functional unity with nuclear reactions. This may be due to the fact that the membranes of the reticulum are structurally linked with the nuclear envelope.

#### Complex SL synthesis

The blue cluster contains reaction parameters associated with the molecular composition of the outer membrane. Since the cell membrane is the biggest reservoir of sphingolipids, particularly complex sphingolipids such as sphingomyelin and glycosphingolipids, this cluster has a strong influence on the overall balance of cellular sphingolipids. The reactions from the pathway of CER production via SM hydrolysis (which was mentioned above) are localized in this cluster. This was confirmed by local sensitivity analysis, the reaction catalyzed by aSMase in the outer membrane has a strong influence on the stability of several modeled species. As a consequence, the pathways responsible for the synthesis of complex sphingolipids (SM and GSL) are localized in this cluster. These reactions strongly affect the stability of endoplasmic CER and subsequently, cytoplasmatic Sph. The blue cluster is comparable to the C2 compartment as denoted by Rao et al. [65] in that it reflects complex SL synthesis. However, extending the results given by Rao et al. [65], we show that it is in functional unity with the reactions of the outer cell membrane. It is worth highlighting that the results of our simulations are consistent with literature reports because SM metabolism in the plasma membrane is known to have strong implications for the balance of bioactive sphingolipids [66].

#### Sphingolipds degradation

The yellow cluster is related to the degradation of complex sphingolipids in the acidic environment of the lysosome. It includes the starting point of the *salvage pathway*, i.e. SM transport and degradation in the lysosome and, to some extent, it resembles the C3 compartment described in [65]. According to the local sensitivity analysis, two reactions from this cluster that represent the transport of SM from the outer membrane to the lysosome and ceramide synthesis from SM in the lysosome may affect the concentration of different molecular species in the model, such as: the lysosomal and outer membrane CER, SM and Sph, endoplasmic CER or mitochondrial S1P and Sph. However, this influence is not very strong. This finding may be explained by the relatively low activity of the lysosomal degradation pathway in cells that develop in favorable conditions. It should be mentioned that according to the clustering analysis, the lysosome belongs to the intersection of the yellow and blue clusters. This seems biologically appropriate because this organelle links the synthesis and degradation pathways of complex SL.

#### Inner membrane balance

The black cluster reflects the molecular balance of the inner membrane and contains reaction parameters that are not mutually related to other compartments, but have a specific effect on the behavior of other pathways. For instance, this cluster contains the reaction catalyzed by nSMase in the inner membrane which, on the basis of local sensitivity analysis, appears to slightly impact the stability of CER, Sph and S1P in the entire model.

### Application of the model: case study of Alzheimer’s disease

Our model not only represents the functional integration of experimental data but may also be used for the computational verification of molecular changes known to cause various human diseases. In recent studies, it has become evident that sphingolipids play important roles in the trafficking and metabolism of AD - related proteins. Thus, these are now acknowledged as crucial molecules in the etiology of AD [23, 67]. This devastating neurodegenerative disorder is characterized by the accumulation of intraneuronal and extracellular protein aggregates and progressive synapse loss. The pathological hallmarks of AD include the extracellular deposition of a peptide called *β*-amyliod (A *β*), and neurofibrillary tangles. The inability to catabolize aggregates of abnormally folded A *β* leads to neuronal degeneration and a subsequent decline in cognitive processes. On the level of sphingolipid metabolism, the most frequently reported hallmarks of the disease are ceramide accumulation in the endoplasmic reticulum and lysosome, and sphingosine accumulation in the cytoplasm accompanied by decreased levels of cytoplasmic S1P and C1P [21–25].

Interestingly, A *β* has been reported to induce increase in CER level through activation of nSMase, resulting in nerve cell death. On the other hand, CER has been shown to alter amyloid-precursor protein processing and A *β* production. This mechanism was described as a CER driven circulus vitiosus where increasing CER level leads to an intensified A *β* production, whereupon A *β* is responsible for CER accumulation [24]. In this study, we applied our model to determine whether changes in enzymatic activity described by Rao et al. [65] lead to expected changes in the concentration of observed SL species.

Currently our model allows to predict the fluctuations in the concentrations of the sphingolipid species and the activity of the enzymes involved in sphingolipid metabolism. Such analysis could be useful to investigate lipidomics aspect of development of various disease. Whereas present application of the model is limited to analyze sphingolipid metabolism as a separate pathway, we plan to integrate our model with genome-scale metabolic network.

#### Computational simulation of Alzheimer’s disease

In an attempt to simulate the cellular response to metabolic disturbances of the SL pathway described in [65], the values of selected reaction parameters necessary to achieve cell homoeostasis were changed. Detailed information is provided in Additional file 1: Table S3. We modified the parameters associated with ceramidase (CDase) activity as well as the parameters corresponding to the dynamics of sphingosine kinase (SK) and ceramide kinase (CERK). Moreover, due to the down-regulation of CERT expression we inhibited the transport of CER to the Golgi apparatus. On the other hand, ceramide *de novo* synthesis (represented by the inflow of CER into the endoplasmic reticulum) was up-regulated. To test the system’s response in an AD scenario we simulated the time evolution of species concentrations.

Preliminary simulations showed that when changes were limited to those described by Rao et al. [65], some modeled species quickly diverged to infinity. Namely, we observed an unexpected, rapid accumulation of SM in several cellular compartments (i.e. the lysosome, outer membrane and endoplasmic reticulum). Another unforeseen system behavior was an increased rate of CER to GSL conversion in the Golgi apparatus, followed by the accumulation of GSL. Since such events do not occur in AD cells, we suggested that the initially introduced modifications should be accompanied by: (i) reduced transport of CER to the Golgi apparatus via a CERT independent pathway, and (ii) increased activity of sphingomyelinases (SMase). We also introduced some minor changes in SM transport between compartments. Our predictions were confirmed by literature reports whereby impaired SM metabolism is known to be linked with AD [68, 69]. These findings emphasize the predictive value of our model.

Once these biologically justified modifications were made, our results were coherent with experimental data: we observed the accumulation of ceramides in cellular compartments, particularly in the endoplasmic reticulum (ER) and lysosome relative to homoeostasis levels [65, 67].

**Fig. 5:**
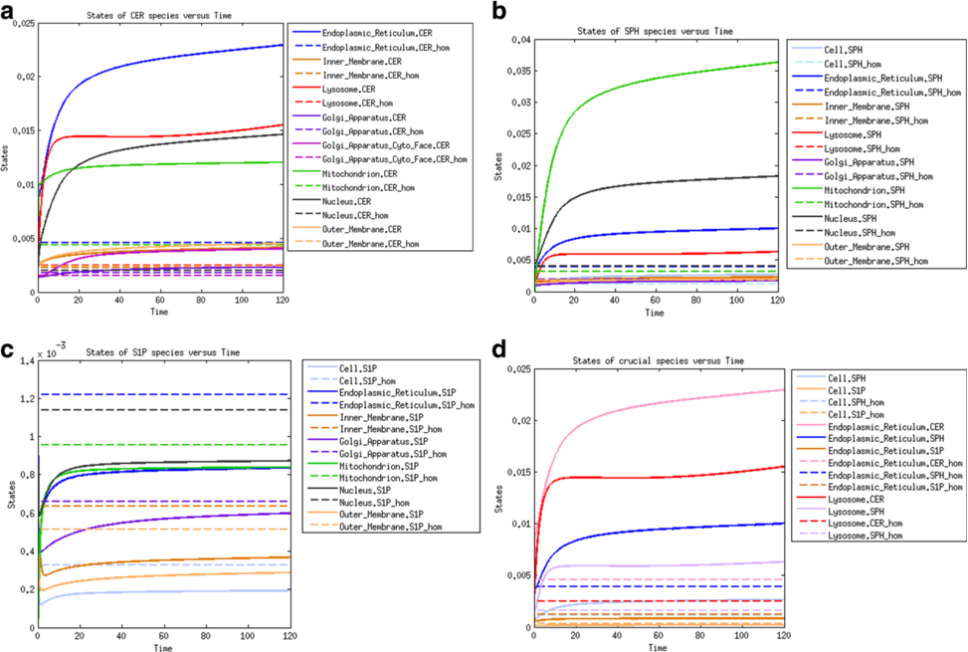
Time evolution of molar concentrations of the following species (the dashed lines correspond to the homeostasis scenario and solid lines to the AD scenario): (**a**) ceramide species; (**b**) sphingosine species; (**c**) sphingosine-1-phosphate species; (**d**) species functionally related to AD

As far as the concentration of Sph species in the model output is concerned, an immediate decline was observed due to CDase down-regulation. This was followed by the accumulation of Sph species in all compartments due to increased concentrations of CER species, the substrates for Sph synthesis. We also observed decreased concentrations of S1P species in the AD scenario (Fig. 5).

#### Local sensitivity analysis: AD scenario vs. homeostasis

Application of the AD scenario yielded slight changes in local sensitivity parameters.

- For the CER species, in contrary to homoeostasis, the most sensitive become ceramides in nucleus and endoplasmic reticulum, which are sensitive basically to endogenous CER in endoplasmic reticulum and exogenous C1P, CER, Sph and S1P in outer membrane inflow parameters as well as nSMase reaction rate in outer membrane.
- The S1P in cytosol becomes sensitive to the SK2 in cytosol, analogously S1P in inner membrane becomes sensitive to SK1 in inner membrane and S1P in outer membrane is more sensitive to inflow parameter of exogenous S1P in outer membrane. However, the mitochondrial S1P becomes invariant to parameters changes.
- Sph species remains largely unchanged with most sensitive mitochondrial S1P.
- Similarly SM species show an unchanged sensitivity with the dominant species SM in outer membrane as most sensitive.

#### Parameter clustering: AD scenario vs. homeostasis

**Fig. 6:**
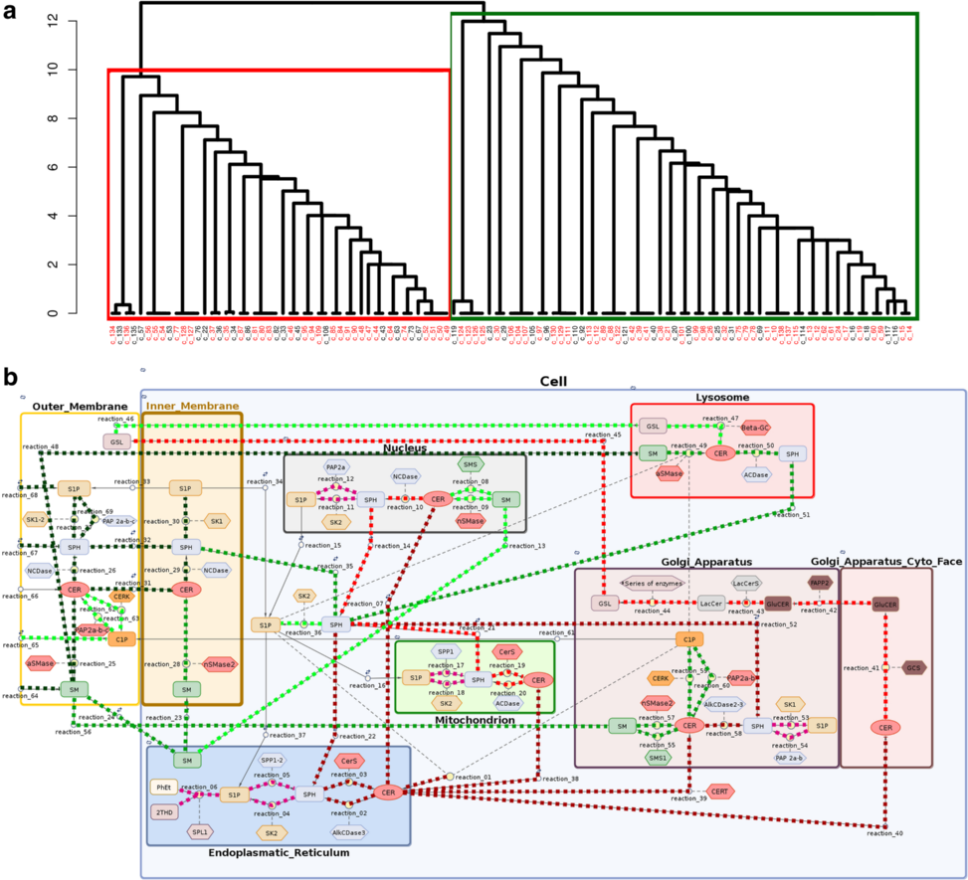
**a** Dendrogram obtained for AD scenario by hierarchical clustering of parameters based on their functional redundancy. Model contains 36 non-identifiable parameters. **b** Clusters of reactions induced by the hierarchical grouping

Clustering analysis of AD model resulted in new parameter dendrogram, with only two clusters in comparison to four clusters obtained in homoeostasis (Fig. 6 vs Fig. 4). Clusters distinguished in AD simulation can be described as follows.

#### Complex sphingolipid metabolism

Parameters that are strongly associated with the metabolism of complex sphingolipids and glycosphingolipids were included in the green cluster. Our results show that changes in SM balance are of great importance for cellular metabolism in AD. This cluster includes ceramide formation via SM hydrolysis, and the catabolism of SM and GSL in the lysosome (*salvage pathway*). Similarly to the results obtained for the state of homoeostasis, simulations in the AD scenario confirm that the hydrolysis of SM in the cell membrane and lysosome strongly influence the level of cytoplasmic Sph. This cluster also includes the parameters related to the synthesis of GSL and SM in the Golgi apparatus. To conclude, this cluster can be viewed as a combination of the green, yellow and part of the blue homeostatic clusters and is formed as a result of increased SM transport and degradation during neurodegeneration.

#### Ceramide synthesis and accumulation

The red cluster includes reactions affected by the inflow of ceramides from the *de novo* synthesis pathway. According to [70], the endoplasmic accumulation of ceramides from this source is an important step in AD development. Clustering analysis confirmed a strong correlation between *de novo* synthesis and concentration of CER in the endoplasmic reticulum and subsequently in the mitochondrion, nucleus and Golgi apparatus.

### Predictive power of the model

In this section we explore the issue of experimental validation of our model. We decided to propose first comprehensive model of sphingolipid metabolism in unspecified human tissue being aware of the scarcity of experimental data. Previous models were based on yeast and mouse datasets and were more specific, see e.g. [27] that models the C16-branch of sphingolipid metabolism in RAW264.7 cells. On the other hand most datasets for human samples come from mass spectrometric analyses of complex body fluids [71]. Such lipidomics data would be of crucial importance while studying the secretion of SL species to these fluids. However for this kind of analysis the intracellular model should be designed first. We would like to emphasize, that almost all model parameters were based on experimental measurements. Particularly the rates for transport reactions were estimated according to experimental data stored in LIPID MAPS database.

Summarizing, the predictive power of our model can be assessed only in a qualitative way, as there are no experimental data available to whose it can be fitted. Therefore we argue, that the computational analysis that reproduces the outcomes from [65] at the moment is the only method available to verify the suitability of the model. Of course, the experimental validation of model predictions would be a subject of follow up research. Currently our collaborators from Mossakowski Medical Institute PAS carry out experiments on SH-SY5Y cell lines and we hope that obtained data would be useful to evaluate the predictive power of our model.

## Conclusions

In the present study, an original model for sphingolipid metabolism in non-specified human tissue was proposed. To the best of our knowledge, this is the most comprehensive model thus far and also the first to explicitly comprise compartmentalization. What is important, we have managed to achieve balance between the complexity and biological soundness of the model and its computational tractability.

Our results demonstrate that this model is an excellent tool to predict the response of the SL pathway to perturbations in the activity of particular enzymes as well as the up- or downregulation of the modeled species. Therefore, the model is perfectly suited to simulate molecular behavior in various scenarios as in this case study of AD.

Moreover, the implementation of semi-independent compartments allows more subtle manipulations of the reaction parameters for specific organelles. Finally, our model enables not only the integration but also validation of experimental data by verifying their cross-compliance in a complex network of interactions.

In addition, the computational validation of the model was performed using recently proposed, sophisticated approaches [31, 32]. Mathematically elegant methods of variance decomposition and sensitivity clustering of parameters revealed non-trivial biological outcomes. Furthermore, the application of the abovementioned approaches in our model served as the perfect validation of their usefulness in realistic size problems.

## Methods

All molecular reactions within a system of interacting species *S*_1_…*S*_*N*_ may be presented in the following manner:

$$R_{j} \colon \qquad \underline{\nu}_{1j} S_{1} + \dots + \underline{\nu}_{Nj} S_{N} \stackrel{k_{j}} \longrightarrow \overline{\nu}_{1j} S_{1} + \dots + \overline{\nu}_{Nj} S_{N}, $$

where $\underline {\nu }_{\textit {nj}}$ and $\overline {\nu }_{\textit {nj}}$ denote amounts of molecules of *n*-th species that are respectively substrate and product of this reaction and the coefficient *k*_*j*_ denotes reaction rate (speed) of the reaction.

**The Mass Action Law kinetics** In case of non-enzymatic transport kinetics we used the Mass Action Law (MAL) principle. The time derivative of each species concentration is the sum of in- and out-fluxes of all neighboring reactions. Here the one reaction flux is equal $k_{j} \left [S_{1}\right ]^{\underline {\nu }_{1j}} \cdot \dots \cdot \left [S_{N}\right ]^{\underline {\nu }_{\textit {Nj}}}$. Hence, ODEs derived from the MAL can be expressed as follows:

$$ \frac{d[S_{n}]}{dt} = \sum\limits_{j=1}^{R} s_{nj} k_{j} \left[S_{1}\right]^{\underline{\nu}_{1j}} \cdot \dots \cdot \left[S_{N}\right]^{\underline{\nu}_{Nj}} \qquad n=1 \dots N $$

where $s_{\textit {nj}} = \overline {\nu }_{\textit {nj}} - \underline {\nu }_{\textit {nj}}$ denotes a stoichiometric coefficient of *n*-th species in *j*-th reaction and [*S*_*n*_] denotes the concentration of *n*-th species.

**The Michaelis Menten kinetics**

Majority of the reactions depicted in the diagram 1 are enzymatic reactions. For this kind of reactions we used Michaelis Menten model (MM) and simplified kinetics derived by the MM model:

$$ \frac{d[P]}{dt}=\frac{V_{max}[S]}{K_{m}+[S]}, $$

where *P* denotes reaction product, *S* denotes reaction substrate and *V*_*max*_, *K*_*m*_ are constant reaction parameters.

**Ordinary differential equations**

Equivalently the ODEs can be expressed in the matrix form:

$$ \frac{d\mathbf{S}(t)}{dt} = M \mathbf{v}(\mathbf{S}(t)), $$

where the system state is represented by the time dependent state vector **S**(*t*) of species concentration, *M* denotes the stoichiometry matrix and **v**(**S**(*t*)) denotes a vector of reaction fluxes (in our model, according to MAL or MM kinetics including inhibition rates) [30].

### Local sensitivity analysis

Local sensitivity analysis shows how the uncertainty of parameters of the model can influence the model output. Sensitivity may be measured by monitoring changes in the output by e.g. partial derivatives of the modeled species to the single parameters. This appears to be a logical approach because any change observed in the output will unambiguously be due to the single variable changed. To compare the sensitivity of the model to the single parameters we constructed the sensitivity indices by time integration of partial derivatives:

$$s_{n,i}={\int_{0}^{T}}\left\vert\frac{\partial S_{n}(t)}{\partial \theta_{i}}\right\vert_{\theta=\theta_{0}}dt $$

where *S*_*n*_ are different species concentrations, *θ* is the vector of parameters and *θ*_0_ is some fixed point in parameters space.

### Variance decomposition

The deterministic approach that represents the mean behavior of the system can also be generalized to a stochastic mode by meaning of Stochastic Differential Equations (SDE), both of which can be represented in a discrete Markov Chain or a continuous Markov Process. Below we sketch the method of variance decomposition as presented by Komorowski et al. [31].

#### Stochastic differential equations

Modeling the system behavior in a stochastic manner means the examination of not only the evolution of the average system state that represents a possible trajectory, but examination of the evolution of the probability distribution over all possible system states.

The most popular approach to describe discrete stochastic model of biochemical pathway is Chemical Master Equation (Chapman-Kolmogorov equation of Markov chain modeling the evolution of the system):

$$ \frac{p P(\mathbf{x},t)}{dt} = \sum\limits_{j}a_{j}(\mathbf{x}-\mathbf{m}_{j})P(\mathbf{x}-\mathbf{m}_{j},t) - \sum\limits_{j}a_{j}(\mathbf{x})P(\mathbf{x},t), $$

where the system state is denoted by the vector $\mathbf {X}(t)\in \mathbb {N}^{N}$ of numbers of molecules each row for one of *N* reacting species, **m**_*j*_ denotes the *j*-th column of stoichiometry matrix *M*=(**m**_1_,…,**m**_*R*_) and *P*(**x**,*t*) denotes the time- and state-dependent distribution of system being in state **X**(*t*)=**x** and finally *a*_*j*_(**X**(*t*)) denotes the propensity function associated with the *j*-th reaction [30].

One of the possible simplifications of the above equation is Linear Noise Approximation, where the dynamic is modeled with Poisson process:

$$\mathbf{X}(t) = \mathbf{X}(0) + \sum\limits_{j = 1}^{R} {\mathbf{m}_{j}}{N_{j}}\left({{\int\limits_{0}^{t}} {{f_{j}}(\mathbf{X}(\tau),\tau)d\tau}} \right) $$

where *N*_*j*_(**X**(*t*),*t*) denotes Poisson process dependent on time and a system state **X**(*t*), corresponding to occurrence of *j*-th reaction. The probability that *j*-th reaction occur during the time interval [*t*;*t*+*dt*) equals *f*_*j*_(*x*,*t*)*dt*, where the *f*_*j*_(*x*,*t*) is called the transition rate.

Although accurate discrete models describe the exact evolution of probability distribution of the system with the assumption that in one time point at most one reaction can occur, they are computational not efficient, as simulations require significant resources. Consequently, it is more efficient to transit from discrete to continuous process. Starting from deterministic approximation:

$$\Phi(t) = \Phi(0) + \sum\limits_{j = 1}^{R} {{m_{j}} {{\int\limits_{0}^{t}} {{f_{j}}(\Phi(s),s)ds} }} $$

where *Φ*(*t*) is the mean system state being the solution of the ODEs that can describe the system state evolution by dividing it into deterministic and stochastic part:

$$ x(t) = \xi(t)+ \Phi(t) $$

where *Φ*(*t*) is the deterministic part and *ξ*(*t*) is the Winer process describing stochastic noise of a system state [72]. The next step of stochastic noise decomposition is to divided noise linearly into noise steaming from separate reactions. The fact, that the total variance:

$$\Sigma(t)=\langle (x(t) - \langle x(t)\rangle) (x(t) - \langle x(t)\rangle)^{T} \rangle $$

is described by the differential equation

(1) $$ \frac{d\Sigma}{dt}={{A}}(t)\Sigma+ \Sigma{{A}}(t)^{T} + {{D}}(t),  $$

where

$$\left\{ A(\Phi,t) \right\}_{ik}=\sum\limits_{j=1}^{r} m_{ij} \frac{\partial f_{j}(\Phi,t)}{\partial \Phi_{k}} $$

and *D*(*t*) denotes diffusion matrix, can be represented as the sum of individual contributions,

(2) $$ \Sigma(t)=\Sigma^{(1)}(t)+\ \ldots\ +\Sigma^{(r)}(t).  $$

results directly from the decomposition of the diffusion matrix ${{D}}(t)=\sum _{j=1}^{r} {{D}}^{(j)}(t)$ and the linearity of the equation for *Σ*(*t*). Komorowski et al. [31] By decomposing variance into the components from individual reactions, we are able to determine the variability that the model has from each reaction, and therefore we are able to assess and weigh the uncertainty of the model in division into single reactions.

### Parameter clustering

Nienałtowski et al. [32] proposed the concept of *functional redundancy* and used it as a dissimilarity measure in a hierarchical clustering algorithm. Let us define the model in Bayesian approach by the distribution of data $(X \in \mathbb {R}^{k})$ given parameters $(\theta \in \mathbb {R}^{l})$ as *P*(*X*|*θ*), together with *a priori* distribution *P*(*θ*). Let us assume that *θ*=(*θ*_*A*_,*θ*_*B*_) corresponds to the division of parameters in two independent sets, then [73]:

(3) $$\begin{array}{*{20}l} H(X) = I(X, \theta_{A}) + I(X, \theta_{B}) + I(\theta_{A}, \theta_{B}| X) + H(X|\theta), \end{array} $$

where *H* denotes here the entropy and *I* is the mutual information between random variables. Here, *I*(*θ*_*A*_,*θ*_*B*_|*X*) measures the part of entropy that is shared by both sets of parameters and is equivalent to redundant knowledge of the model, which is owned by *θ*_*A*_ and *θ*_*B*_.

The computation of *I*(*θ*_*A*_,*θ*_*B*_|*X*) requires calculation integral over all possible outcomes of the model, which is highly inefficient; hence, this notion was replaced with the local redundancy measure, which substitute assumption of knowledge regarding the model *X* with information regarding initial parameters *θ*^∗^. Thus, functional redundancy is equal to *I*(*θ*_*A*_,*θ*_*B*_|*θ*^∗^) and is calculated according to a given formula [74]:

(4) $$\begin{array}{@{}rcl@{}} I(\theta_{A}, \theta_{B}|\theta^{*}) = -\frac{1}{2}\sum\limits_{i = 1}^{\text{min}(|\theta_{A}|, |\theta_{B}|)} \text{log}(1 - {\rho_{j}^{2}}), \end{array} $$

where *ρ*_*i*_ stands for the canonical correlation obtained from the Fisher information matrix of *θ*^∗^ (*FIM*(*θ*^∗^)).

Moreover, to indicate *non-identifiable* parameters, the authors defined (*δ*,*ζ*) - identifiability using the idea of functional redundancy [32]. In this terminology, *θ*_*i*_ is (*δ*,*ζ*) – identifiable if *FIM*_*ii*_(*θ*)>*ζ* and *ρ*(*θ*_*i*_,*θ*_−*i*_)<1−*δ*, where *θ*_−*i*_ represents all parameters except *θ*_*i*_.

Using functional redundancy, we can cluster parameters according to a hierarchical algorithm (i.e. in every turn of the loop we merge two sets of parameters with the biggest redundancy measure and remove all non-indentifiable parameters from further analysis) and visualize it on a dendrogram.

## Note

SMBL file with the model implementation of homoeostasis and model implementation of AD are provided as Additional file 2 and Additional file 3.

## Additional files

Additional file 1
**Supplementary Figures and Tables.**

Additional file 2
**SMBL file with the model implementation of homoeostasis.**

Additional file 3
**SMBL file with the model implementation of AD.**
