# Supplementary material for: Computational modeling of sphingolipid metabolism
Source: BMC Syst Biol. 2015 Aug 15;9:47. doi: 10.1186/s12918-015-0176-9 (PMC4537549; doi:10.1186/s12918-015-0176-9)
Supplement: Additional file 1 — Supplementary Figures and Tables. [file 12918_2015_176_MOESM1_ESM.pdf]

Computational modeling of sphingolipids metabolism

Agata Charzyńska♠,[1,2], Weronika Wronowska♠,[3] , Karol Nienałtowski[4] , Anna Gambin*[5,2]

[1]Institute of Computer Science Polish Academy of Sciences, Warsaw, Poland;
[2]Bioinformatics Laboratory, Mossakowski Medical Research Centre Polish Academy of Sciences, Warsaw, Poland;
[3]Faculty of Biology University of Warsaw, Warsaw, Poland;
[4]Division of Modelling in Biology and Medicine, Institute of Fundamental Technological Research Polish Academy of Sciences, Warsaw, Poland;
[5]Institute of Informatics, University of Warsaw, Warsaw, Poland;

Email: a.charzynska@phd.ipipan.waw.pl; wwro@biol.uw.edu.pl; k.nienaltowski@sysbiosig.org; aniag@mimuw.edu.pl;

*Corresponding author

Supplementary Figures

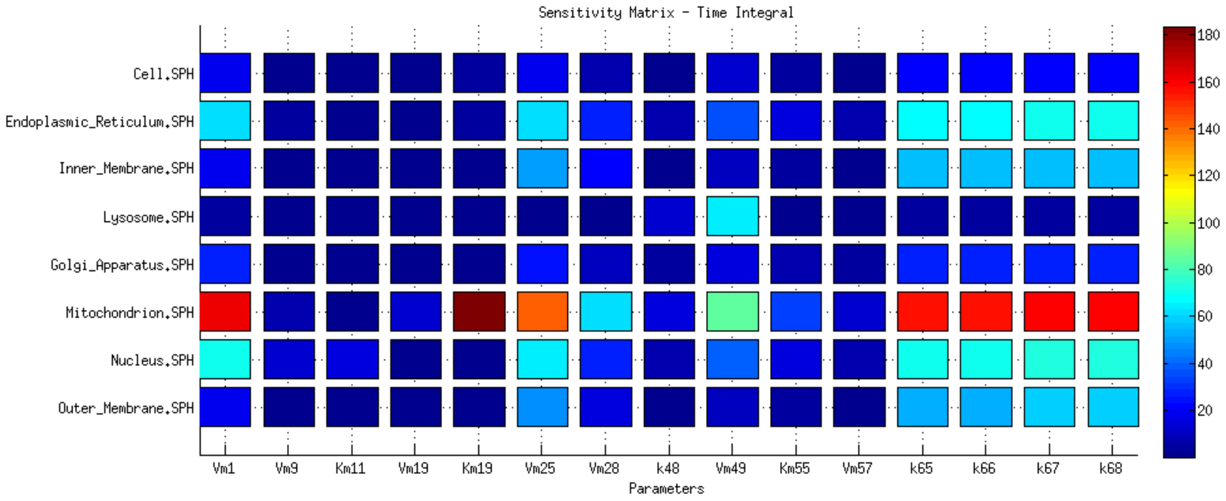

Figure 1: The local sensitivity analysis of the SPH species to the highly significant parameters.

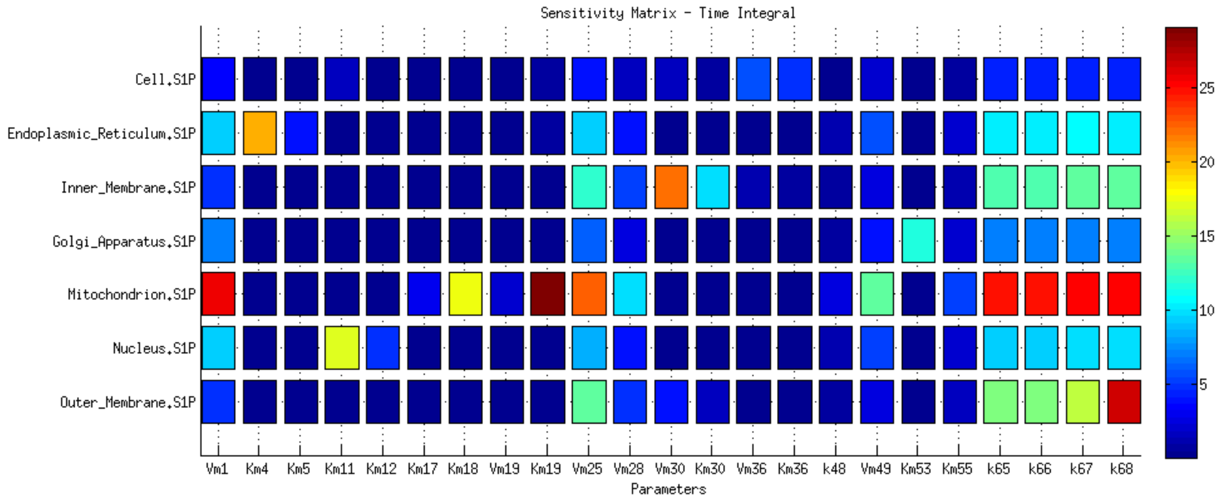

Figure 2: The local sensitivity analysis of the S1P species to the highly significant parameters.

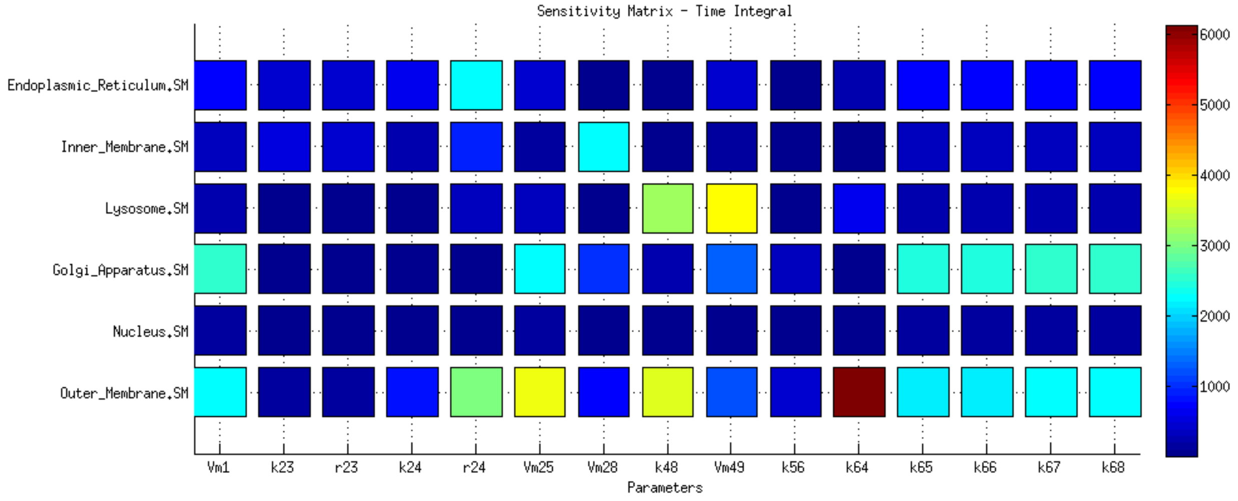

Figure 3: The local sensitivity analysis of the SM species to the highly significant parameters.

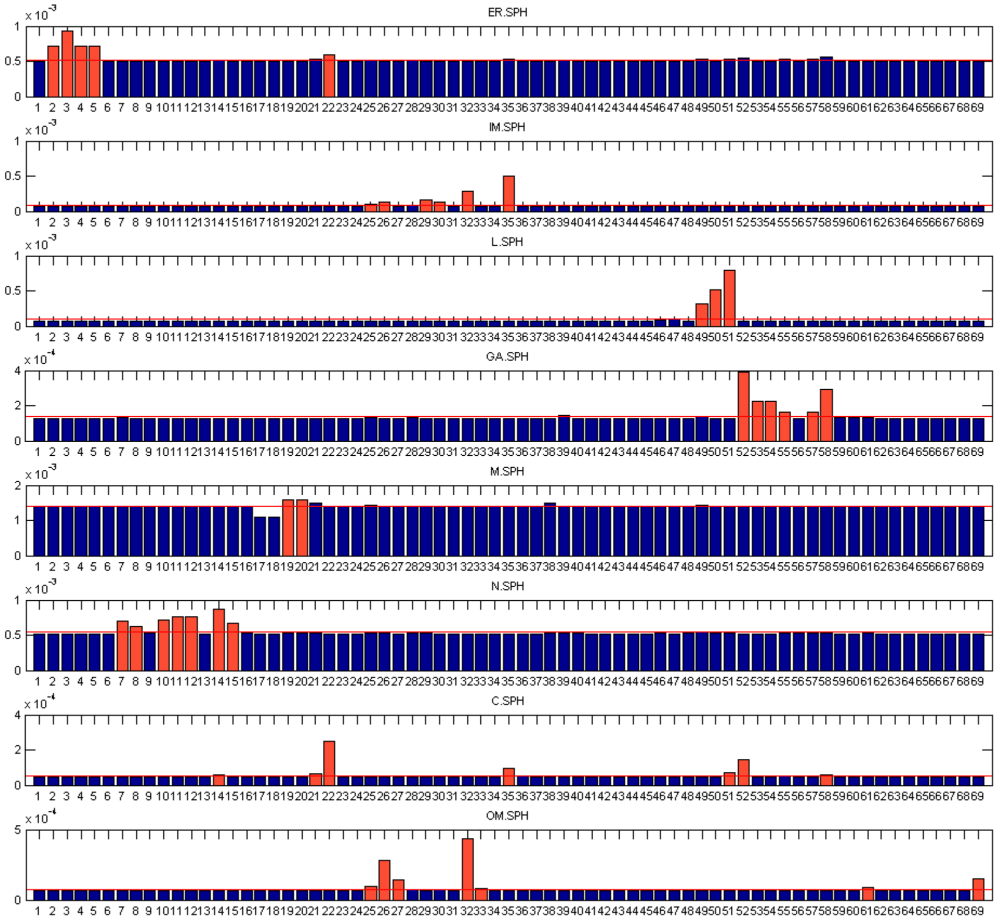

Figure 4: The variance decomposition of the sphingosine concentration into components steaming from all model reactions. The red lines denotes the averages variance components of the investigated species. The red bars denotes the variance components that exceed the threshold of 110% of average.

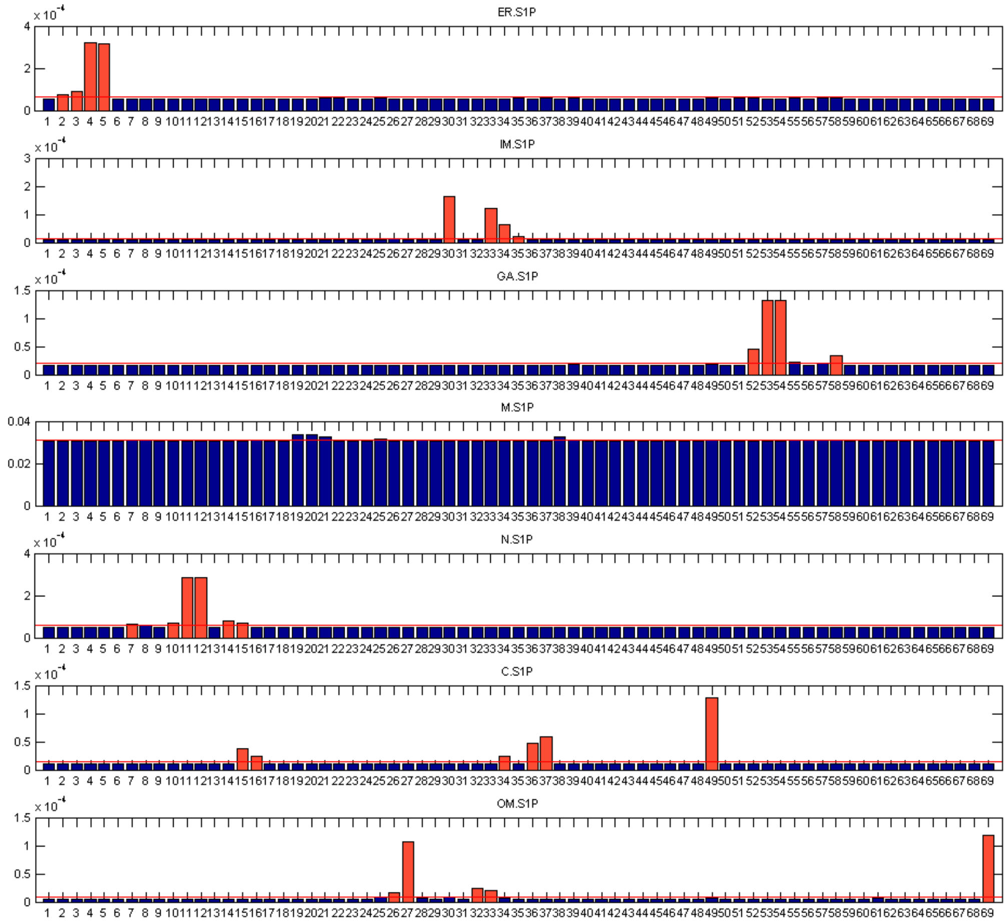

Figure 5: The variance decomposition of the sphingosine-1-phosphate concentration into components steaming from all model reactions. The red lines denotes the averages variance components of the investigated species. The red bars denotes the variance components that exceed the threshold of 110% of average.

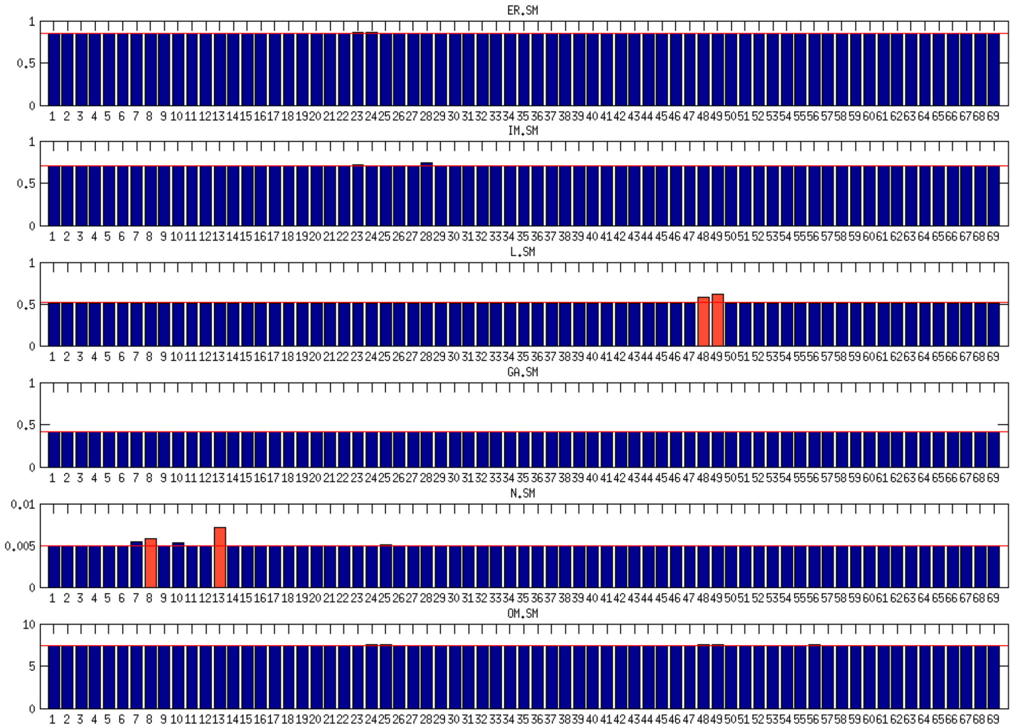

Figure 6: The variance decomposition of the sphingomyeline concentration into components steaming from all model reactions. The red lines denotes the averages variance components of the investigated species. The red bars denotes the variance components that exceed the threshold of 110% of average.

Reaction no.	Compartment	Reaction Substrate	Reaction Product	Kinetics	Reaction Flux	Enzyme Transporter	Km (nmol/mg)	Vm (nmol/min mg)	k (1/min)	r (1/min)	Literature Km (nmol/mg)	Literature Vm (nmol/min mg)	Source Km	Source Vm
1	ER	-	ER.CER	MAL & Inhibition	$\frac{Vm1}{(1+C.S1P/Ki_{S1P})*(1+GA.C1P/Ki_{C1P})}$	de novo synthesis	-	0	-	-	-	-		
2	ER	ER.CER	ER.SPH	MM	$\frac{Vm2*ER.CER}{Km2+ER.CER}$	AlkCDase3	0,081	0,48	-	-	0,081	0,27	[1]	[1]
3	ER	ER.SPH	ER.CER	MM	$\frac{Vm3*ER.SPH}{Km3+ER.SPH}$	CerS	0,171	2,4	-	-	0,171	11,3	[2]	[2]
4	ER	ER.SPH	ER.S1P	MM	$\frac{Vm4*ER.SPH}{Km4+ER.SPH}$	SK2	0,0034	0,175	-	-	0,0034	0,175	[3]	[3]
5	ER	ER.S1P	ER.SPH	MM	$\frac{Vm5*ER.S1P}{Km5+ER.S1P}$	SPP1-2	0,0385	3,1	-	-	0,0385	36,4	[4]	[4]
6	ER	ER.S1P	PhEt +2THD	MM	$\frac{Vm6*ER.SPH}{Km6+ER.SPH}$	SPL1	0,035	0	-	-	0,035	-	[5]	
7	ER-N	ER.CER	N.CER	MAL	$k7 * ER.CER - r7 * N.CER$	-	-	-	0,8	0,1	-	-		
8	N	N.CER	N.SM	MM	$\frac{Vm8*N.CER}{Km8+N.CER}$	SMS	0,155	0,109	-	-	0,155	0,109	[6]	[6]
9	N	N.SM	N.CER	MM	$\frac{Vm9*N.SM}{Km9+N.SM}$	nSMase	0,126	0,00113	-	-	0,126	0,113	[6]	[6]
10	N	N.CER	N.SPH	MM	$\frac{Vm10*N.CER}{Km10+N.CER}$	NCDase	0,0601	0,068	-	-	0,0601	0,68	[7]	[7]
11	N	N.SPH	N.S1P	MM	$\frac{Vm11*N.S1P}{Km11+N.S1P}$	SK2	0,0034	0,1	-	-	0,0034	0,1	[3]	[3]
12	N	N.S1P	N.SPH	MM	$\frac{Vm12*N.SPH}{Km12+N.SPH}$	PAP2a	0,025	1,24	-	-	0,025	124	[8]	[8]
13	N-ER	N.SM	ER.SM	MAL	$k13 * N.SM - r13 * ER.SM$	-	-	-	0,12	0,001	-	-		
14	N-C	N.SPH	C.SPH	MAL	$k14 * N.SPH - r14 * C.SPH$	-	-	-	0,5	0,23	-	-		
15	N-C	N.S1P	C.S1P	MAL	$k15 * N.S1P - r15 * C.S1P$	-	-	-	0,44	0,25	-	-		
16	C-M	C.S1P	M.S1P	MAL	$k16 * C.S1P - r16 * M.S1P$	-	-	-	0,25	0,044	-	-		
17	M	M.S1P	M.SPH	MM	$\frac{Vm17*M.S1P}{Km17+M.S1P}$	SPP1	0,0385	3	-	-	0,0385	36,4	[4]	[4]
18	M	M.SPH	M.S1P	MM	$\frac{Vm18*M.SPH}{Km18+M.SPH}$	SK2	0,0034	0,15	-	-	0,0034	0,18	[3]	[3]
19	M	M.SPH	M.CER	MM	$\frac{Vm19*M.CER}{Km19+M.CER}$	CerS	0,0025	0,1	-	-	0,0025	0,0714	[9]	[9]
20	M	M.CER	M.SPH	MM	$\frac{Vm20*M.CER}{Km20+M.CER}$	ACDase	0,149	2,27	-	-	0,149	2,27	[10]	[10]
21	M-C	M.SPH	C.SPH	MAL	$k21 * M.SPH - r21 * C.SPH$	-	-	-	0,43	0,034	-	-		
22	C-ER	C.SPH	ER.SPH	MAL	$k22 * C.SPH - r22 * ER.SPH$	-	-	-	23	0,03	-	-		
23	ER-IM	ER.SM	IM.SM	MAL	$k23 * ER.SM - r23 * IM.SM$	-	-	-	0,01	0,005	-	-		
24	ER-OM	ER.SM	OM.SPH	MAL	$k24 * ER.SM - r24 * OM.SM$	-	-	-	0,006	0,001	-	-		
25	OM	OM.SM	OM.CER	MM	$\frac{Vm25*OM.SM}{Km25+OM.SM}$	aSMase	0,0455	0,001	-	-	0,0455	0,183	[6]	[6]
26	OM	OM.CER	OM.SPH	MM	$\frac{Vm26*OM.CER}{Km26+OM.CER}$	NCDase	0,0601	0,12	-	-	0,0601	0,68	[7]	[7]
27	OM	OM.SPH	OM.S1P	MM	$\frac{Vm27*OM.SPH}{Km27+OM.SPH}$	SK1-2	0,034	0,11	-	-	0,0034	0,1		
28	IM	IM.SM	IM.CER	MM	$\frac{Vm28*IM.SM}{Km28+IM.SM}$	nSMase2	0,148	0,002	-	-	0,148	0,344	[6]	[6]
29	IM	IM.CER	IM.SPH	MM	$\frac{Vm29*IM.CER}{Km29+IM.CER}$	NCDase	0,0601	0,03	-	-	0,0601	0,68	[7]	[7]

Table 1: **Table of reactions and parameters of the sphingolipids metabolism model with parameters in steady state - homestasis level**

Reaction no.	Compartment	Reaction Substrate	Reaction Product	Kinetics	Reaction Flux	Enzyme Transporter	Km (nmol/mg)	Vm (nmol/min mg)	k (1/min)	r (1/min)	Literature Km (nmol/mg)	Literature Vm (nmol/min mg)	Source Km	Source Vm
30	IM	IM.SPH	IM.S1P	MM	$\frac{Vm30*IM.SPH}{Km30+IM.SPH}$	SK1	0,00506	0,003	-	-	0,00506	0,0565	[11]	[11]
31	IM-OM	IM.CER	OM.CER	MAL	$k31*IM.CER-r31*OM.CER$	-	-	-	1	1	-	-		
32	IM-OM	IM.SPH	OM.SPH	MAL	$k32*IM.SPH-r32*OM.SPH$	-	-	-	1	3	-	-		
33	IM-OM	IM.S1P	OM.S1P	MAL	$k33*IM.S1P$	-	-	-	1	-	-	-		
34	IM-C	IM.S1P	C.S1P	MAL	$k34*IM.S1P-r34*C.S1P$	-	-	-	0,4	0,25	-	-		
35	IM.C	IM.SPH	C.SPH	MAL	$k35*IM.SPH – r35*C.SPH$	-	-	-	3	0,0269				
36	C	C.SPH	C.S1P	MM	$\frac{Vm36*C.S1P}{Km36+C.S1P}$	SK2	0,0034	0,0036	-	-	0,0034	0,036	[3]	
37	C-ER	C.S1P	ER.S1P	MAL	$k37*C.S1P-r37*ER.S1P$	-	-	-	4,5	0,005	-	-		
38	ER-M	ER.CER	M.CER	MAL	$k38*ER.CER-r38*M.CER$	-	-	-	1	0,75	-	-		
39	ER-GA	ER.CER	GA.CER	MAL	$\frac{Vm39*ER.CER}{Km39+ER.CER}$	CERT	-	-	5	-	-	-		
40	ER-GACF	ER.CER	GAOL. CER	MAL	$k40*ER.CER$	-	-	-	0,08	-	-	-		
41	GACF	GAOL. CER	GACF. GluCER	MM	$\frac{Vm41*GAOL.CER}{Km41+GAOL.CER}$	GCS	0,04	0,01	-	-	0,04	10	[12]	
42	GACF-GA	GAOL. GluCER	GA. GluCER	MAL	$\frac{Vm38*GAOL.GluCER}{Km38+GAOL.GluCER}$	FAPP2	-	-	1	-	-	-		
43	GA	GA. GluCER	GA. LacCer	MM	$\frac{Vm43*GA.GluCER}{Km43+GA.GluCER}$	LacCerS	0,003	0,001	-	-	0,003	0,001	[13]	[13]
44	GA	GA. LacCER	GA.GSL	MM	$\frac{Vm44*GA.LacCer}{Km44+GA.LacCer}$	Series of enzymes	0,003	0,001	-	-	0,003	0,001		
45	GA-OM	GA.GSL	OM.GSL	MAL	$k45*GA.GSL$	-	-	-	0,2	-	-	-		
46	OM-L	OM.GSL	L.GSL	MAL	$k46*OM.GSL$	-	-	-	0,03	-	-	-		
47	L	L.GSL	L.CER	MM	$\frac{Vm47*L.GSL}{Km47+L.GSL}$	Beta-GC	0,019	0,0061	-	-	0,019	0,0061	[14]	[14]
48	OM.L	OM.SM	L.SM	MAL	$k48*OM.SM$	-	-	-	0,002	-	-	-		
49	L	L.SM	L.CER	MM & Inhibition	$\frac{Vm49*L.SM}{(Km49+L.SM)*(1+GA.C1P/Kic1p)*(1+C.S1P/Kis1p)}$	aSMase	0,0455	0,00183	-	-	0,0455	0,183	[6]	[6]
50	L	L.CER	L.SPH	MM	$\frac{Vm50*L.CER}{Km50+L.CER}$	ACDase	0,149	0,1	-	-	0,149	2,27	[10]	[10]
51	L-C	L.SPH	L.SPH	MAL	$k51*L.SPH$	-	-	-	1	-	-	-		
52	C-GA	C.SPH	GA.SPH	MAL	$k52*C.SPH – r52*GA.SPH$	-	-	-	0,2	9	-	-		
53	GA	GA.SPH	GA.S1P	MM	$\frac{Vm53*GA.SPH}{Km53+GA.SPH}$	SK1	0,00506	0,13	-	-	0,00506	0,0565	[11]	[11]
54	GA	GA.S1P	GA.SPH	MM	$\frac{Vm54*GA.S1P}{Km54+GA.S1P}$	PAP 2a-b	0,036	2	-	-	0,036	10	[8]	[8]
55	GA	GA.CER	GA.SM	MM	$\frac{Vm55*GA.CER}{Km55+GA.CER}$	SMS1	0,02	0,3	-	-	4,6	0,14	[6]	[6]
56	GA-OM	GA.SM	OM.SM	MAL	$k56*GA.SM$	-	-	-	0,015	-	-	-		

Table 1: **Table of reactions and parameters of the sphingolipids metabolism model with parameters in steady state - homestasis level**

Reaction no.	Compartment	Reaction Substrate	Reaction Product	Kinetics	Reaction Flux	Enzyme Transporter	Km (nmol/mg)	Vm (nmol/min mg)	k (1/min)	r (1/min)	Literature Km (nmol/mg)	Literature Vm (nmol/min mg)	Source Km	Source Vm
57	GA	GA.SM	GA.CER	MM	$\frac{Vm57*GA.SM}{Km57+GA.SM}$	nSMase2	0,148	0,05	-	-	0,148	0,344	[6]	[6]
58	GA	GA.CER	GA.SPH	MM	$\frac{Vm58*GA.CER}{Km58+GA.CER}$	AlkCDase2-3	0,081	0,8	-	-	0.081	0,027	[1]	[1]
59	GA	GA.CER	GA.C1P	MM	$\frac{Vm59*GA.CER}{Km59+GA.CER}$	CERK	0,107	2	-	-	0,107	21	[15]	[15]
60	GA	GA.C1P	GA.CER	MM	$\frac{Vm60*GA.C1P}{Km60+GA.C1P}$	PAP2a-b	0,036	0,75	-	-	0,036	10	[8]	[8]
61	GA-OM	GA.C1P	OM.C1P	MAL	$k61*GA.C1P$	-	-	-	2,5	-	-	-		
62	OM	OM.C1P	OM.CER	MM	$\frac{Vm62*OM.CER}{Km62+OM.CER}$	PAP2a-b-c	0,036	0,78	-	-	0,036	10	[8]	[8]
63	OM	OM.CER	OM.C1P	MM	$\frac{Vm63*OM.C1P}{Km63+OM.C1P}$	CERK	0,107	2,1	-	-	0,107	21	[15]	[15]
64	OM	-	OM.SM	MAL	$k64$	-	-	-	0	-	-	-		
65	OM	-	OM.C1P	MAL	$k65-r65*OM.C1P$	-	-	-	0	0	-	-		
66	OM	-	OM.CER	MAL	$k66$	-	-	-	0	-	-	-		
67	OM	-	OM.SPH	MAL	$k67-r67*OM.SPH$	-	-	-	0	0	-	-		
68	OM	-	OM.S1P	MAL	$k68-r68*OM.S1P$	-	-	-	0	0	-	-		
69	OM	OM.S1P	OM.SPH	MM	$\frac{Vm69*OM.S1P}{Km69+OM.S1P}$	PAP 2a-b-c	0,036	0,43	-	-	0,036	10		

Table 1: **Table of reactions and parameters of the sphingolipids metabolism model with parameters in steady state - homestasis level**

No.	Species	Initial concentrations (nmol/mg)
1	GA.C1P	0,0015
2	OM.C1P	0,0025
3	ER.CER	0,0046
4	IM.CER	0,0023
5	L.CER	0,0025
6	GA.CER	0,0018
7	GACF.CER	0,0015
8	M.CER	0,0044
9	N.CER	0,002
10	OM.CER	0,0024
11	GA.GluCER	0,0017
12	GACF.GluCER	0,0037
13	L.GSL	0,0012
14	GA.GSL	0,0018
15	OM.GSL	0,0122
16	GA.LacCer	0,0017
17	Cell.S1P	0,0003
18	ER.S1P	0,0012
19	IM.S1P	0,0006
20	GA.S1P	0,0007
21	M.S1P	0,001
22	N.S1P	0,0011
23	OM.S1P	0,0005
24	ER.SM	0,1613
25	IM.SM	0,1331
26	L.SM	0,1002
27	GA.SM	0,1234
28	N.SM	0,0121
29	OM.SM	0,6287
30	Cell.SPH	0,0012
31	ER.SPH	0,0039
32	IM.SPH	0,0019
33	L.SPH	0,0016
34	GA.SPH	0,0019
35	M.SPH	0,0032
36	N.SPH	0,0041
37	OM.SPH	0,0018

Table 2: Initial values of species in stationary state - homeostasis level

Reaction no.	Compartment	Reaction Substrate	Reaction Product	Enzyme Transporter	AD scenario Km (nmol/mg)	AD scenario Vm (nmol/min mg)	AD scenario k (1/min)	AD scenario r (1/min)
1	ER	-	ER.CER	de novo synthesis		0,004		
2	ER	ER.CER	ER.SPH	AlkCDase3	0,081	0,32		
3	ER	ER.SPH	ER.CER	CerS	0,171	2,4		
4	ER	ER.SPH	ER.S1P	SK2	0,0034	0,0875		
5	ER	ER.S1P	ER.SPH	SPP1-2	0,0385	3,1		
6	ER	ER.S1P	PhEt+2THD	SPL1	0,035	0		
7	ER-N	ER.CER	N.CER	-			0,8	0,100
8	N	N.CER	N.SM	SMS	0,155	0,109		
9	N	N.SM	N.CER	nSMase	0,126	0,1		
10	N	N.CER	N.SPH	NCDase	0,0601	0,0453		
11	N	N.SPH	N.S1P	SK2	0,0034	0,05		
12	N	N.S1P	N.SPH	PAP2a	0,025	1,24		
13	N-ER	N.SM	ER.SM	-			0,12	0,0010
14	N-C	N.SPH	C.SPH	-			0,5	0,2300
15	N-C	N.S1P	C.S1P	-			0,44	0,2500
16	C-M	C.S1P	M.S1P	-			0,25	0,0440
17	M	M.S1P	M.SPH	SPP1	0,0385	3		
18	M	M.SPH	M.S1P	SK2	0,0034	0,07		
19	M	M.SPH	M.CER	CerS	0,0025	0,1		
20	M	M.CER	M.SPH	ACDase	0,149	2		
21	M-C	M.SPH	C.SPH	-			0,43	0,03400
22	C-ER	C.SPH	ER.SPH	-			23	0,03000
23	ER-IM	ER.SM	IM.SM	-			0,01	0,00500
24	ER-OM	ER.SM	OM.SPH	-			0,015	0,00100
25	OM	OM.SM	OM.CER	aSMase	0,0455	0,002		
26	OM	OM.CER	OM.SPH	NCDase	0,0601	0,08		
27	OM	OM.SPH	OM.S1P	SK1-2	0,034	0,055		
28	IM	IM.SM	IM.CER	nSMase2	0,148	0,0025		
29	IM	IM.CER	IM.SPH	NCDase	0,0601	0,02		
30	IM	IM.SPH	IM.S1P	SK1	0,00506	0,0015		
31	IM-OM	IM.CER	OM.CER	-			1	1,00000
32	IM-OM	IM.SPH	OM.SPH	-			0,01	3,00000
33	IM-OM	IM.S1P	OM.S1P	-			1	
34	IM-C	IM.S1P	C.S1P	-			0,4	0,25000
35	IM.C	IM.SPH	C.SPH	-			3	0,02690
36	C	C.SPH	C.S1P	SK2	0,0034	0,001		
37	C-ER	C.S1P	ER.S1P	-			4,5	0,0050
38	ER-M	ER.CER	M.CER	-			1	0,7500
39	ER-GA	ER.CER	GA.CER	CERT			1	
40	ER-GACF	ER.CER	GAOL.CER	-			0,02	
41	GACF	GAOL.CER	GACF.GluCER	GCS	0,04	0,01		
42	GACF-GA	GAOL.GluCER	GA.GluCER	FAPP2			0,1	
43	GA	GA.GluCER	GA.LacCer	LacCerS	0,003	0,001		
44	GA	GA.LacCER	GA.GSL	Series of enzymes	0,003	0,001		
45	GA-OM	GA.GSL	OM.GSL	-			0,2	
46	OM-L	OM.GSL	L.GSL	-			0,03	
47	L	L.GSL	L.CER	Beta-GC	0,019	0,0061		
48	OM.L	OM.SM	L.SM	-			0,011	
49	L	L.SM	L.CER	aSMase	0,0455	0,01		
50	L	L.CER	L.SPH	ACDase	0,149	0,00669		
51	L-C	L.SPH	L.SPH	-			1	
52	C-GA	C.SPH	GA.SPH	-			0,2	9,000000
53	GA	GA.SPH	GA.S1P	SK1	0,00506	0,08		
54	GA	GA.S1P	GA.SPH	PAP 2a-b	0,036	2		
55	GA	GA.CER	GA.SM	SMS1	0,02	0,3		
56	GA-OM	GA.SM	OM.SM	-			0,045	
57	GA	GA.SM	GA.CER	nSMase2	0,148	0,07		
58	GA	GA.CER	GA.SPH	AlkCDase2-3	0,081	0,529		
59	GA	GA.CER	GA.C1P	CERK	0,107	0,5		
60	GA	GA.C1P	GA.CER	PAP2a-b	0,036	0,75		
61	GA-OM	GA.C1P	OM.C1P	-			2,5	
62	OM	OM.C1P	OM.CER	PAP2a-b-c	0,036	0,78		
63	OM	OM.CER	OM.C1P	CERK	0,107	0,5		
64	OM	-	OM.SM	-			0	
65	OM	-	OM.C1P	-			0	0
66	OM	-	OM.CER	-			0	
67	OM	-	OM.SPH	-			0	0
68	OM	-	OM.S1P	-			0	0
69	OM	OM.S1P	OM.SPH	PAP 2a-b-c	0,036	0,43		

Table 3: Parameters in Alzheimer's Disease scenario. Red parameters are down regulated, green parameters are up regulated in compare to homeostasis level

Additional files

SBML Ceramide_methabolism_homeostaza.xml: SMBL file with the homoeostasis model.

SBML Ceramide_methabolism_AD.xml: SMBL file with the model implementation of AD.

References

1. Sun W, Jin J, Xu R, Hu W, Szulc ZM, Bielawski J, Obeid LM, Mao C: **Substrate specificity, membrane topology, and activity regulation of human alkaline ceramidase 2 (ACER2)**. *J. Biol. Chem.* 2010, **285**(12):8995–9007.
2. Shimeno H, Soeda S, Sakamoto M, Kouchi T, Kowakame T, Kihara T: **Partial purification and characterization of sphingosine N-acyltransferase (ceramide synthase) from bovine liver mitochondrion-rich fraction**. *Lipids* 1998, **33**(6):601–605.
3. Liu H, Sugiura M, Nava VE, Edsall LC, Kono K, Poulton S, Milstien S, Kohama T, Spiegel S: **Molecular cloning and functional characterization of a novel mammalian sphingosine kinase type 2 isoform**. *J. Biol. Chem.* 2000, **275**(26):19513–19520.
4. Le Stunff H, Peterson C, Thornton R, Milstien S, Mandala SM, Spiegel S: **Characterization of murine sphingosine-1-phosphate phosphohydrolase**. *J. Biol. Chem.* 2002, **277**(11):8920–8927.
5. Bandhuvula P, Li Z, Bittman R, Saba JD: **Sphingosine 1-phosphate lyase enzyme assay using a BODIPY-labeled substrate**. *Biochem. Biophys. Res. Commun.* 2009, **380**(2):366–370.
6. Albi E, Cataldi S, Bartoccini E, Mazzoni F, Voccoli V, Viola Magni M, Lazzarini R, Garcia-Gil M: **Sphingomyelin metabolism changes after serum deprivation in neuronal cell nuclei**. In *Proceedings of the 8th meeting of Sphingolipid Club, the Association of the Sphingolipidologists: 14-16 November 2010; Glasgow.* Edited by Club S, Association of the Sphingolipidologists 2010:80.
7. Galadari S, Wu BX, Mao C, Roddy P, El Bawab S, Hannun YA: **Identification of a novel amidase motif in neutral ceramidase**. *Biochem. J.* 2006, **393**(Pt 3):687–695.
8. Alderton F, Darroch P, Sambi B, McKie A, Ahmed IS, Pyne N, Pyne S: **G-protein-coupled receptor stimulation of the p42/p44 mitogen-activated protein kinase pathway is attenuated by lipid phosphate phosphatases 1, 1a, and 2 in human embryonic kidney 293 cells**. *J. Biol. Chem.* 2001, **276**(16):13452–13460.
9. Laviad EL, Albee L, Pankova-Kholmyansky I, Epstein S, Park H, Merrill AH, Futerman AH: **Characterization of ceramide synthase 2: tissue distribution, substrate specificity, and inhibition by sphingosine 1-phosphate**. *J. Biol. Chem.* 2008, **283**(9):5677–5684.
10. Bernardo K, Hurwitz R, Zenk T, Desnick RJ, Ferlinz K, Schuchman EH, Sandhoff K: **Purification, characterization, and biosynthesis of human acid ceramidase**. *J. Biol. Chem.* 1995, **270**(19):11098–11102.
11. Melendez AJ, Carlos-Dias E, Gosink M, Allen JM, Takacs L: **Human sphingosine kinase: molecular cloning, functional characterization and tissue distribution**. *Gene* 2000, **251**:19–26.
12. Gupta V, Patwardhan GA, Zhang QJ, Cabot MC, Jazwinski SM, Liu YY: **Direct quantitative determination of ceramide glycosylation in vivo: a new approach to evaluate cellular enzyme activity of glucosylceramide synthase**. *J. Lipid Res.* 2010, **51**(4):866–874.
13. Chatterjee S, Castiglione E: **UDPgalactose:glucosylceramide beta 1—-4-galactosyltransferase activity in human proximal tubular cells from normal and familial hypercholesterolemic homozygotes**. *Biochim. Biophys. Acta* 1987, **923**:136–142.
14. Brumshtein B, Salinas P, Peterson B, Chan V, Silman I, Sussman JL, Savickas PJ, Robinson GS, Futerman AH: **Characterization of gene-activated human acid-beta-glucosidase: crystal structure, glycan composition, and internalization into macrophages**. *Glycobiology* 2010, **20**:24–32.
15. Munagala N, Nguyen S, Lam W, Lee J, Joly A, McMillan K, Zhang W: **Identification of small molecule ceramide kinase inhibitors using a homogeneous chemiluminescence high throughput assay**. *Assay Drug Dev Technol* 2007, **5**:65–73.
